
JOURNAL INFORMATION
==============================
NLM Title Abbreviation: ScientificWorldJournal
Journal ID: ScientificWorldJournal
NLM Journal ID: 101131163
Journal ID: TSWJ

The Scientific World Journal

ISSN: 2356-6140
EISSN: 1537-744X
Publisher: Wiley

ARTICLE INFORMATION
==============================
PMCID: PMC5974805
PMID: 29887763
DOI: 10.1100/tsw.2003.43
Article ID: 949780
Article version: 1
Subjects: Editorial

Peer-Reviewed Protocols: A Repository within TheScientificWorldJOURNAL

Allen Anna Vindenes *
Director of Editorial Publishing, TheScientificWorld,
*Anna Vindenes Allen: aallen@thescientificworld.com
Publication date: 2003
Electronic publication date: 2003 Jun 20
Volume: 3
First page: 546
Last page: 557
Copyright: Copyright © 2003 Anna Vindenes Allen.
Copyright year: 2003
License: This is an open access article distributed under the Creative Commons Attribution License, which permits unrestricted use, distribution, and reproduction in any medium, provided the original work is properly cited.
License URL: https://creativecommons.org/licenses/by/3.0/

==============================
Publisher's Note

*TheScientificWorldJOURNAL* (2003) 3, 546–557
ISSN 1537-744X; DOI 10.1100/tsw.2003.43

### Peer-Reviewed Protocols: A Repository within *TheScientificWorldJOURNAL*

Anne Vindenes Allen
*Director of Editorial Publishing, TheScientificWorld*

E-mail: aallen@thescientificworld.com

*Accepted June 9, 2003; Published June 20, 2003*

**The Methods and Protocols Domain of *TheScientificWorldJOURNAL* publishes Peer-Reviewed Protocol articles that add the verification and rigor needed to ensure that the scientific community benefits from best practice techniques. All published articles are indexed by major abstracting services, and linked to the available literature via databases such as sciBASE (http://www.thescientificworld.com/) and Article Finder (http://www.infotrieve.com/). Full Instructions for Authors are included.**

**DOMAINS:** methods and protocols

Progress in science is wholly dependent on experimentation, so it is surprising that many protocols do not make it beyond the pages of a laboratory notebook where they cannot accelerate scientific research in the wider community. Laboratories frequently develop their own protocols or adapt known methods, but so far many of these that have reached publication have often stayed buried within the confines of a research article. As a result, protocols can be difficult to locate by other scientists, which consequently impedes the rate at which our understanding progresses.

It is evident that easy access to clear, comprehensive protocols is paramount, but that published methods must be scientifically sound. A domain of *TheScientificWorldJOURNAL*, Methods and Protocols, has been established to build a repository of Peer-Reviewed Protocol articles, adding the verification and rigor that is needed to ensure that authors and the scientific community as a whole benefits from best practice techniques. Principal Editor Professor Rickwood, of the University of Essex, and his Editorial Board oversee scientific standards and accept for peer-review a range of methods-related articles types, including methods reviews, technical notes, and original research papers.

Judging by responses to past announcements of the publication of Peer-Reviewed Protocols, it is clear that there is significant interest from authors whose expertise covers a broad spectrum of scientific disciplines. Results from a survey by *TheScientificWorldJOURNAL* of methods

©2003 with author.

specialists show that there is a strong need for the publication of these articles and that the move toward peer-review and scientific verification of protocols is well received by scientists, with a large majority of respondents saying that they support the move toward protocol articles being peer-reviewed and published in the citable journal literature. Over 80% of respondents to the survey believe there is also value in peer-review and publication of protocols already freely available on web sites. The majority of scientists agree that peer-reviewed publication helps with sharing of knowledge: Reliable protocols are often hard to find.

Upon acceptance for *TheScientificWorldJOURNAL*, all protocol articles are published within the Methods and Protocols Domain, as well as being associated with all other relevant scientific domains of *TheScientificWorldJOURNAL*. Published protocols are also linked to the available literature via databases such as sciBASE (http://www.thescientificworld.com/) and Article Finder (http://www.infotrieve.com/).

Following close consultation with experts in scientific methodologies, a set of Author Guidelines was developed to ensure a useful and consistent approach to the publication in *TheScientificWorldJOURNAL* Protocol Repository (see appendix below). *TheScientificWorldJOURNAL* has already published many Peer-Reviewed Protocol articles and many more are in the pipeline. A sample article is also available on *TheScientificWorldJOURNAL*'s web site (http://www.thescientificworldjournal.com/).

### ACKNOWLEDGEMENT

TheScientificWorld acknowledges the contribution made by Ms. Claire Redhead in the writing of this article.

**This article should be referenced as follows:**

Vindenes Allen, A. (2003) Peer-Reviewed Protocols: A Repository within *TheScientificWorldJOURNAL*. *TheScientificWorldJOURNAL* **3**, 546–557.

### APPENDIX

### Publishing Methods and Protocols in TheScientificWorldJOURNAL

The provision of high quality information of scientific methods and protocols is one of the most neglected areas of scientific publishing today – yet this information forms the basic building block of scientific experimentation. Scientific protocols must be easily located and accessed, archived, and above all, they must be clear, comprehensive and scientifically sound.

*TheScientificWorldJOURNAL* has addressed this by providing for the publication of Peer-Reviewed Protocol articles, adding the verification and rigor to ensure the scientific community benefits from best practice techniques. Published articles will form part of a rapidly growing repository for high quality scientific methodologies, and linked to bibliographic services, such as MedLine, and also to methodsBASE, the premier digital resource. Submissions and peer-review is overseen by expert Editorial Board Members of TheScientificWorldJOURNAL's *Methods and Protocols* Domain, working in conjunction with other eminent scientists from all Domains of the Journal.

### Related Articles Types - Methods and Protocols

*TheScientificWorldJOURNAL* offers opportunity for publication of other methodology-related articles:

**Methods Paper**

An original research paper focusing on a particular method or group of methods, with discussion on aspects such as the reliability of the methods, its applications, and practical feasibility.

**Technical Note**

An abbreviated form of the Methods Paper of usually less than 4 pages (PDF).

**Review and Mini-Review Articles**

Usually comparing several methods already reported in the literature – the mini-review is less than 12 pages in length and provides a more in-depth topic focus than full review papers.

Instructions to Authors available on request for these related article types.

### GETTING YOUR PAPER REVIEWED AND PUBLISHED EFFECTIVELY

To help *TheScientificWorldJOURNAL* and its Editors process your manuscript effectively, **please follow these guidelines carefully:**

- *TheScientificWorldJOURNAL* does not accept manuscripts previously submitted to or published in other journals.
- All authors are responsible for submitting manuscripts in comprehensible US or UK English and ensuring scientific accuracy.
- Authors are required to agree to the terms of the Author License on submission of their manuscript. This ensures Authors retain copyright on publication, while providing the Publisher the rights to distribute the article worldwide. A copy of the Author Agreement is available on request from tsw@infotrieve.com or through the website at http://www.thescientificworldjournal.com/agreement.asp
- Authors are solely responsible for obtaining permission for the use of figures, tables, illustrations, etc. from other publications and must retain proof that they have done so.
- If your manuscript is accepted subject to revision, you will be expected to revise the manuscript promptly. If there is a significant delay in executing revisions, the manuscript will be regarded as a new submission.

### HOW TO SUBMIT YOUR MANUSCRIPT

Your covering letter should include:

- A **list the scientific Domain(s)** of *TheScientificWorldJOURNAL* to which your manuscript is relevant (see Appendix 1 below). Highlight **one Domain** that is of most relevance.

- Contact details of 5 potential referees, including their email addresses
- Special instructions or notes to the Editor.

All manuscripts, including text and figures, must be submitted in electronic form. When submitting via email, ensure your attachments do not exceed 2 MB total file size, but send several emails if required.

Send your manuscript, figures and covering letter in electronic form to Tara Retzlaff at tretzlaff@infotrieve.com

### HOW TO PREPARE FILES

Ensure to use your name (surname and initials) and date (month/day/year) in the file name:

For text: **smithjp_mss_021803.doc**
For figures: **smithjp_fig3_021803.jpg**
For tables: **smithjp_tbl1_021803.gif**

Complex tables, line drawings, half tones, color images, equations and mathematical formulae should be submitted as graphic images. All graphic files must be submitted in sufficiently high resolution (300 dpi).

Graphics should be submitted individually as separate files. Do not include figure captions or table legends as part of the graphic image. Please ensure that files are not saved as "read only".

### HOW TO PREPARE YOUR MANUSCRIPT

- Manuscripts are to be prepared in the English language.
- For the **main text** body use **Word, WordPerfect, or RTF** formatted documents.
- For **illustrations / images** use **.tif, .gif, .jpg, .eps, .bmp, .xl.**

### Illustrations and images

- Figures must be in FINAL FORM ready for incorporation into HTML files, with a maximum width of 600 pixels.
- Keep in mind that while the use of color is encouraged, manuscripts may be printed by readers in black and white, so the choice of colors should be carefully considered. Cross-hatching and stippling are preferable to shading. Light colors (yellow, light blue etc.) should be avoided.
- Authors are responsible for the quality of the images. Always double check how artwork appears on the screen and how it prints before submitting.
- The individual graphic files must be named with their respective numbers and format types. Be sure to indicate clearly the placement and corresponding file name.

### Text

- It is best to use a clear, easy-to-read typeface/font (e.g., Times Roman, Arial) for text, references, etc.
- Ensure all characters are correctly represented throughout the manuscript, e.g., 1 (one), l (lower case el), I (upper case I), 0 (zero), O (upper case oh), x (ex), × (times symbol).
- Use the appropriate symbol function (e.g., in Word - Insert/Symbol) for Greek letters, mathematical symbols, etc.

- Abbreviations should be spelled out and defined at first mention in the text, then abbreviated thereafter.

- If available in your word processing program, use the hyperlink capability to create internal or external links. *TheScientificWorldJOURNAL* will check links to make sure they operate properly. Authors are responsible for informing the Publisher of any changes to URL's after publication of their articles.

- Links to outside sources:
    If you wish to link from your manuscript to outside sources, you are responsible for the accuracy and relevance of those sites. All the information necessary to make the link should be provided. (For example, to make a link from the reference section directly to a cited article, you must first check that the article is available to users and that the URL is accurately recorded in the citation.)

*For example:* Molecular representations were made using C3#D (http://www.ncbi.nlm.nih.gov/Structure/CN3D/cn3d.shtml)

- Links to references will be made through Infotrieve' s Article Finder® literature database – see http://www.infotrieve.com/

*Other references - example:* Steinberger, M. (1996) Electronic Mathematics Journals, Notices of the American Mathematical Society, **43**, 13-16. URL:http://www.ams.org/notices/199601/steinberger.pdf

### SUBMISSION CONSTITUENTS

Your paper should consist of the following elements:

1. **Title**
2. **Subtitle** (if any)
3. **Author names**
4. **Affiliations**
5. **Email addresses**
6. **Keywords and phrases**
7. **Domains**
8. **Method Types**
9. **Abstract**
10. **Synopsis**
11. **Main text**
12. **Figures, tables, multi-media elements**
13. **Acknowledgements**
14. **References / Bibliography**
15. **Patent Information (Optional)**
16. **Biosketches**

### Author names

The author submitting the manuscript is considered the ***Corresponding Author***. Use an asterisk (*) to indicate the Corresponding Author, e.g. David A. Smith*.

Example:
Bob E. Jones, Jr.,[1] Martin F. Davidson, Jr.,[2] Sam J. Smith,[3] and Trevor M. Olivier[*,1]
[1]*Department of Engineering, University of Anystate, 200 Main St., Anytown, XX 00000 ;*
[2]*Geology Department, Seismic Activity University, 3456 Tremor Street, Unsinkable, CA*

*99999 ; [3] Department of Physiology, Institute of Medicine, 300 Orange, Sunshine, FL 33300*

**E-mail addresses**

Please supply email addresses of all authors. The Publisher will only provide authors' e-mail addresses in connection with *TheScientificWorldJOURNAL* on request.

**Keywords and phrases**

Submit as many keywords and phrases as you consider would be useful in locating your manuscript. The Editors and the Publisher may edit key word list for consistency. Alternative spellings, common abbreviations and alternative names should be provided. For example: Serotonin (5-hydroxytryptamine, 5-HT). These will be stored with the manuscript and will aid retrieval from key word and free text searches.

**Domains**

Select ALL scientific audiences who may have an interest in your research (see Appendix 1). This will help publicize your paper to the wider scientific community. Highlight the Domain that is the principal Domain as this will help in the peer-reviewing process.

**Method Type**

e.g. separation (author may choose from the list below or create own)

Bioassays/Physiological Analysis
Chromatographic Techniques
Crystallographic Techniques
Electrophoretic Techniques
Extraction, Isolation, Purification and Separation Techniques
Histological/Cytological and Culture Techniques
Imaging Techniques
Immunologic Techniques
Invasive/Surgical Techniques
Mathematical and Computer Techniques
Molecular Biology Techniques and Chemical Characterization
Pharmacological/Toxicological Techniques
Preparatory and General Laboratory Techniques
Spectrum Analysis Techniques
Synthetic Techniques

**Abstract**

Abstracts should be concise and to the point, and clearly state the scope and scientific context of the article in language that will be understood by a broad scientific, medical and technical audience.

**Synopsis**

This is a short, promotional statement of no more than 75 words that will be used in conjunction with the publication of your article (for instance, as part of Tables of Contents). It should help explain the broader significance and context of your research. Do not use specialist terms, and do not repeat text found within the abstract.

**Main Text**

Organize your headings and subheadings as follows:

**Introduction** – protocol background

**Materials and equipment** – what you need to prepare for protocol (include all vendor information)

- list of solutions (final concentration, stock solutions, recipe for given volume)
- list of biological resources required
- list of physical requirements (tubes/plates/etc)
- list of general equipment required (cell culture hood/incubators/water baths/stirrers)
- list of equipment to be reserved in advance and for how long (e.g. ultracentrifuge)

**Methods –** what to do and how

- numbered, step-by-step instruction for executing protocol e.g. add this to that mix by inverting/vortexing/stirring spin 5’ @ 1000g tips and troubleshooting information
  e.g. lysed DNA looks like a gooey mass prior to centrifugation
  note any steps that require special attention

**Figures, tables, multi-media elements etc**

Authors may submit extended datasets, tables and figures and enrich content using video, audio, animation and 3-D.

Graphics should be submitted individually as separate files. Be sure to indicate clearly the placement and corresponding file name.

If you have a large number of images available or they are too large for the screen constraints, a link must be made from the manuscript to your home site.

*Multi-media:* If you enhance your manuscript with audio, video, 3D or animation, a link must be made from the manuscript to your home website. The correct reference should be posted and linked to *TheScientificWorldJOURNAL* with a brief description of the contents in the manuscript itself. If you need further assistance, contact us at tsw@infotrieve.com.

**Acknowledgments**

Unless otherwise specified, acknowledgement will be given to *TheScientificWorldJOURNAL* Editor responsible for handling your manuscript. We recommend including details of any relevant grants, including grant reference numbers, as well as details of relevant patents.

**References / Bibliography**

Authors are responsible for the accuracy of the references and must make sure all references cited within the text are also listed in the Reference List. There is no limit placed on the number of references in any manuscript. References are to be numbered consecutively in the text and the reference section using the following styles:

**TEXT:**

"This theory is supported by vaccine studies using a gene gun to administer plasmid. It has been demonstrated that, after intradermal administration of plasmid with a gene gun, plasmid directly transfects the resident APC of the skin (Langerhans cells), resulting in the subsequent migration of these cells to the draining lymph node, where antigen is expressed [12]."

**REFERENCE SECTION:**

12. Condon, C., Watkins, S.C., Celluzzi, C.M., Thompson, K., and Falo, Jr., L.D. (1996) DNA-based immunization by in vivo transfection of dendritic cells. *Nat. Med.* 2, 1122-1128.

**Patent Information (Optional)**

You may include the following information if you wish:

Patent Number
Patent Date
Patent Class
Patent Assignee
Patent Country

**Biosketches**

Authors are invited to submit a brief biosketch of credentials. Biosketches appear after the Reference section of the article.

*Example:*

Gary D. Hickman is a Principal Environmental Scientist with the Tennessee Valley Authority in Norris, Tennessee. He received B.S. and M.S. degrees from the University of Arkansas and is a Certified Fisheries Scientist by the American Fisheries Society.

### HOW TO CITE YOUR PAPER

Your paper should be cited using the following format:

Colgan, S. P., Comeford, K. M., and Lawrence, D. W. (2002) Epithelial Cell-Neutrophil Interactions in the Alimentary Tract: A Complex Dialog in Mucosal Surveillance and Inflammation, *TheScientificWorldJOURNAL*, Vol. 2, 76-88. URL:http://www.thescientificworldJOURNAL.com

### NEED HELP?

At any time, you may contact us with questions: tretzlaff@infotrieve.com or tsw@infotrieve.com.

### APPENDIX II

### Publishing Domains of TheScientificWorldJOURNAL

Please review the listing below and select ALL of the publishing Domains that are of relevance to your article. You can select as many Domains as are relevant, but please indicate which Domain is the principal Domain as this will assist with the peer-review process. Domain selection is subject to peer-review.

Your work will be promoted to all of our members that have indicated an interest in the fields your article covers. For your convenience, the Domains have been divided into general areas of research interest (e.g. Genetics, Genomics & Proteomics). Please make sure that you look at all of these generalized research areas when searching for relevant Domains.

### GENETICS, GENOMICS & PROTEOMICS

- Bioinformatics
- DNA Replication
- DNA Repair
- Enzymology & Protein - Protein Interaction
- Evolutionary Genetics
- Gene Expression
- Genetic Engineering
- Genetics (fly)
- Genetics (man)
- Genetics (mouse)
- Genetics (plants)
- Genetics (worms)
- Genetics (yeast)
- Genetics (zebrafish)
- Genomics
- Gene Therapy
- Molecular Engineering
- Molecular Evolution
- Pharmacogenomics
- Protein Databases
- Protein Degradation
- Protein Engineering
- Protein Folding
- Protein Synthesis
- Protein Trafficking
- Protein Transcription
- Protein Transport
- Proteomics
- Statistical Genetics
- Toxicogenomics
- Transcription & Gene Regulation
- Xenopus Genetics

### PHARMACOLOGY & PHARMACEUTICS

- Bioanalysis
- Cell Therapy
- Clinical Pharmacology
- Drug Adherence
- Drug Delivery
- Drug Dependence
- Drug Design
- Drug Discovery
- Drug Metabolism
- Drug Receptor
- Drug Transport
- High Throughput Screening
- Gene Therapy
- Molecular Modeling
- Molecular Pharmacology
- Pharmaceutical Analysis
- Pharmaceutical Sciences
- Pharmacodynamics
- Pharmacognosy
- Molecular Modeling
- Pharmacokinetics
- Pharmacokinetics & Pharmacodynamics Modeling
- Pharmacology
- Psychopharmacology
- Receptor Pharmacology
- Therapeutic Drug Modeling
- Vaccine Discovery

### CELL BIOLOGY

- Cell & Tissue Culture
- Cell Biology
- Cell Cycle
- Cell Cycle Meiosis
- Cell Cycle Mitosis
- Endothelial Function
- Extracellular Matrix
- Glial Function
- Intracellular Signaling
- Membrane Channel Function

- Cell Death
- Cell Fate & Determination
- Cell Therapy
- Cell and Tissue Differentiation
- Neuronal Function
- Patterning
- Plant Cell Biology
- RNA Metabolism

### PHYSIOLOGY & PATHOPHYSIOLOGY PROCESSES

- Aging
- Atherosclerosis
- Biological Transport
- Cognition
- Developmental Biology
- Growth & Growth Factors
- Higher Level Brain Function
- Infection
- Inflammation
- Lymphokines & Chemokines
- Metabolic Disease
- Metabolism
- Metastasis
- Microbial Pathogenesis
- Motility
- Motor Processes
- Neural Networks
- Oncology
- Pulmonary Pathology
- Rheumatology
- Sensation & Perception
- Skeletal Diseases
- Synapse Formation
- Thrombosis
- Toxicology
- Trans Membrane Signaling

### PHYSIOLOGY & PATHOPHYSIOLOGY SYSTEMS

- Bone Biology
- Cardiovascular Biology
- Embryology
- Endocrinology
- Gastroenterology
- Hematology
- Immunology
- Microbial Pathogenesis
- Microbiology (Archaeal Biology)
- Microbiology (General and Applied)
- Microbiology (Bacteriology)
- Microbiology (Fungal Biology)
- Microbiology (Parasitology)
- Nephrology
- Neurology
- Neuroscience
- Pathology
- Pulmonology
- Reproduction
- Urology
- Virology

### METHODS IN PHYSIOLOGY & PATHOPHYSIOLOGY

- Computational Biology
- Forensic Science
- Methods and Protocols
- Microscopy
- Modeling
- Structural Biology
- Virtual Experimentation

### BIOLOGICAL SCIENCES

- Aquatic Toxicology
- Animal Behavior
- Animal Reproduction
- Animal Sciences
- Biochemistry
- Bioenergetics
- Bioengineering
- Biomaterials
- Biomimetics
- Biophysics
- Biotechnology
- Botany
- Entomology
- Fermentation
- Invertebrate Zoology
- Molecular Biology
- Plant Processes
- Plant Sciences
- Plant Taxonomy
- Signaling
- Taxonomy & Systematics
- Vertebrate Zoology
- Veterinary Microbiology
- Veterinary Pathology
- Veterinary Pharmacology
- Veterinary Science

### FOOD SCIENCE & TECHNOLOGY

- Agricultural Biotechnology
- Agronomy
- Crop Science
- Dairy Science
- Food Engineering
- Food Microbiology
- Genetically Modified Foods
- Meat Science
- Nutrition

### CHEMISTRY

- Analytical Chemistry
- Chemical Engineering
- Chemical Methods
- Clinical Chemistry
- Combinatorial Chemistry
- Glycoscience
- Green Chemistry
- Inorganic Chemistry
- Medicinal Chemistry
- Natural Products Chemistry
- Organic Chemistry
- Pesticide Chemistry
- Physical &Theoretical Chemistry

### SOCIAL & BEHAVIOURAL SCIENCES

- Anthropology
- Behavior
- Behavioral Psychology
- Human Rights
- Learning and Memory
- Philosophy of Science
- Psychiatry
- Social Psychology
- Sociology

### HEALTH CARE SCIENCES

- Anesthesiology
- Clinical Medicine
- Child Health and Human Development
- Clinical Psychology
- Clinical Trials
- Complementary & Alternative Medicine
- Dermatology
- Ear, Nose and Throat
- Emergency Medicine
- Experimental Medicine
- Gynecology
- Imaging
- Medical Care
- Medical Care (men's health)
- Medical Care (women's health)
- Medical Education
- Medical Ethics
- Medical Informatics
- Medical Research
- Musculoskeletal Disorders
- Nursing
- Opthalmology
- Palliative Care
- Sports Science & Physiotherapy
- Surgery
- Tissue Engineering

### ENVIRONMENT & ECOSYSTEMS

- Atmospheric Systems
- Biodiversity
- Biomarkers
- Bioremediation & Bioavailability
- Chemical Composition
- Coastal Systems
- Cold Climate Environmental Systems
- Ecosystems & Communities
- Ecosystems Management
- Environmental Chemistry
- Environmental Genomics
- Environmental Endocrine Disruptors
- Environmental Management & Policy
- Environmental Microbiology
- Environmental Modeling
- Environmental Monitoring
- Environmental Mutagens
- Environmental Systems and Tracers
- Environmental Technology
- Forestry
- Freshwater Systems
- Global Systems
- Heavy Metals In The Environment
- Invasive Species
- Isotopes in the Environment
- Life Cycle Assessment
- Marine Systems
- Oceans Biology
- Oceans Chemistry
- Oceans Physics
- Persistent Organic Pollutants
- Pesticide Residues
- Plant Ecology
- Risk & Impact Assessment
- Soil Systems
- Terrestrial Environmental Toxicology
- Terrestrial Systems
- Tropical Systems
- Waste Management Policy
- Water Science & Technology
